# Effect of exenatide on the pharmacokinetics of a combination oral contraceptive in healthy women: an open-label, randomised, crossover trial

**DOI:** 10.1186/1472-6904-12-8

**Published:** 2012-03-19

**Authors:** Prajakti A Kothare, Mary E Seger, Justin Northrup, Kenneth Mace, Malcolm I Mitchell, Helle Linnebjerg

**Affiliations:** 1 Eli Lilly and Company, Lilly Corporate Center, Indianapolis, IN, USA; 2 Eli Lilly and Company, Lilly Research Center, Earl Wood Manor, Windlesham, Surrey GU20 6PH, UK

**Keywords:** exenatide twice daily, pharmacokinetics, oral contraceptive

## Abstract

**Background:**

Consistent with its effect on gastric emptying, exenatide, an injectable treatment for type 2 diabetes, may slow the absorption rate of concomitantly administered oral drugs resulting in a decrease in maximum concentration (C_max_). This study evaluated the drug interaction potential of exenatide when administered adjunctively with oral contraceptives, given their potential concomitant use.

**Methods:**

This trial evaluated the effect of exenatide co-administration on single- and multiple-dose pharmacokinetics of a combination oral contraceptive (ethinyl estradiol [EE] 30 μg, levonorgestrel [LV] 150 μg [Microgynon 30^®^]). Thirty-two healthy female subjects participated in an open-label, randomised, crossover trial with 3 treatment periods (oral contraceptive alone, 1 hour before exenatide, 30 minutes after exenatide). Subjects received a single dose of oral contraceptive on Day 8 of each period and QD doses on Days 10 through 28. During treatment periods of concomitant usage, exenatide was administered subcutaneously prior to morning and evening meals at 5 μg BID from Days 1 through 4 and at 10 μg BID from Days 5 through 22. Single- (Day 8) and multiple-dose (Day 22) pharmacokinetic profiles were assessed for each treatment period.

**Results:**

Exenatide did not alter the bioavailability nor decrease daily trough concentrations for either oral contraceptive component. No substantive changes in oral contraceptive pharmacokinetics occurred when oral contraceptive was administered 1 hour before exenatide. Single-dose oral contraceptive administration 30 minutes after exenatide resulted in mean (90% CI) C_max _reductions of 46% (42-51%) and 41% (35-47%) for EE and LV, respectively. Repeated daily oral contraceptive administration 30 minutes after exenatide resulted in C_max _reductions of 45% (40-50%) and 27% (21-33%) for EE and LV, respectively. Peak oral contraceptive concentrations were delayed approximately 3 to 4 hours. Mild-to-moderate nausea and vomiting were the most common adverse events observed during the trial.

**Conclusions:**

The observed reduction in C_max _is likely of limited importance given the unaltered oral contraceptive bioavailability and trough concentrations; however, for oral medications that are dependent on threshold concentrations for efficacy, such as contraceptives and antibiotics, patients should be advised to take those drugs at least 1 hour before exenatide injection.

**Trial registration:**

ClinicalTrials.gov: NCT00254800.

## Background

Exenatide, a 39-amino acid peptide and antidiabetic agent known as a glucagon-like peptide-1 receptor agonist, has multiple glucoregulatory actions which are similar to those of endogenous glucagon-like peptide-1. In the European Union, it is an adjunctive therapy for patients with type 2 diabetes who are suboptimally controlled with metformin, a sulphonylurea, a thiazolidinedione, and combinations of metformin plus a sulphonylurea or metformin plus a thiazolidinedione. In the United States, exenatide is indicated as an adjunct to diet and exercise to improve glycaemic control in adults with type 2 diabetes mellitus. Following a subcutaneous dose (5 or 10 μg BID), exenatide is rapidly absorbed with a time to peak concentration (T_max_) of approximately 2 hours, has a terminal half-life (t_1/2_) of 2.4 hours [1], and is predominantly eliminated by passive renal mechanisms [2]. Exenatide has been shown to reduce fasting and postprandial glucose by the combined contribution of glucose-dependent insulin secretion, suppression of glucagon secretion, and slowing of gastric emptying [3-5]. There is evidence that this treatment also reduces appetite [6] and energy intake [7].

Consistent with its pharmacological effect of slowing gastric emptying, exenatide may reduce the rate of absorption of concomitantly administered oral drugs. Drug-drug interaction studies with digoxin [8], warfarin [9], lovastatin [10], and lisinopril [11] have demonstrated that concomitant exenatide treatment reduced the maximum plasma concentrations (C_max_) and delayed the T_max _for these drugs, both of which are consistent with slowing of gastric emptying. Reductions in overall exposure (area under the curve [AUC]) were only observed in the exenatide-lovastatin interaction study. However, given the known pharmacokinetic characteristics of exenatide, the potential for a CYP3A induction was considered unlikely and the observed results were considered to be related to incomplete characterization of the single-dose lovastatin AUC following exenatide dosing. Acetaminophen [12], a marker of gastric emptying, was studied with exenatide to understand how the relative timing of exenatide administration might change the magnitude of pharmacokinetic effects observed for orally administered drugs. In addition, this prior study provided information on the optimal timing for administration of other concomitant oral medications. Changes in the acetaminophen profile were not evident when acetaminophen was given 1 hour prior to the exenatide dose as the absorption process for acetaminophen had likely been completed before the onset of exenatide action. However, a reduced C_max _and delayed T_max _were observed when acetaminophen was given after exenatide administration; the magnitude of changes was greatest 1 to 2 hours after exenatide administration.

The present study evaluated the drug-drug interaction potential of exenatide with a widely used concomitantly administered combination oral contraceptive (OC) consisting of ethinyl estradiol (EE) and levonorgestrel (LV). The study included a treatment period in which OC was administered 30 minutes after exenatide, such that the anticipated time of peak exposure of the oral contraceptive would coincide with the maximum effect of slowed gastric emptying. A second treatment period with OC administered 1 hour before exenatide was also included. No interaction would be expected during the second treatment period, as OC absorption was likely to be completed prior to the onset of exenatide action. The interaction was assessed after single, as well as multiple doses of OC to maximise pharmacokinetic information generated from the study.

## Methods

### Subjects

This study was conducted at 1 clinical study center in the United Kingdom. The protocol was approved by the Independent Ethics Committee Plymouth, UK, and was conducted in accordance with the 1975 Declaration of Helsinki, as revised in 2000 [13], and the European Commission's directive on clinical research (2001/20/CE) [14]. Before enrollment, all subjects provided written informed consent. Subjects were required to be taking an OC prior to study entry and be healthy pre-menopausal females, 18 to 45 years old, with a BMI between 19 to 35 kg/m^2^. Subjects were excluded from the study if they had diabetes mellitus or had received implanted contraceptives for 6 months or injectable contraceptives for 12 months prior to the study. Grapefruit was restricted within 7 days and concomitant drug therapies that could induce or inhibit CYP3A were not permitted within 14 days before the first drug administration. In case of mild intercurrent illness during the study, ibuprofen and/or anti-emetic medications that would not affect gastrointestinal motility were allowed at the discretion of the investigator. Lifestyle habits of eligible subjects, such as smoking, alcohol consumption, diet, and exercise, were not altered during the study.

### Study design

This was an open-label, 3-period, 3-sequence, randomised crossover study in healthy female subjects who were using OCs prior to study entry (clinicaltrials.gov registration: NCT00254800). The primary objective was to evaluate the effect of exenatide on the multiple-dose PK of a combination oral contraceptive (EE and LV) administered 1 hour before and 30 minutes after the exenatide dose. Up to 40 subjects were to be enrolled to ensure that approximately 18 subjects completed the study. Comparing OC alone and OC administered 1 hour before exenatide, a sample size of 18 subjects was estimated to provide approximately 90% power to demonstrate that the 90% CI of the ratio of geometric means for AUC (EE or LV) would be contained within the interval (0.80, 1.25). This sample size estimate was based on an intra-subject coefficient of variation of 15%.

The OC combination product (Microgynon 30^®^) consisted of EE, 30 μg and LV, 150 μg. Prior to starting the active dosing period, screening was conducted over 2 visits. The purpose of the first screening visit, approximately 2 months prior to admission, was to initiate a run-in period either to convert to the study OC or to synchronise the OC cycle within a cohort of subjects. The second screening visit occurred approximately 21 days prior to the first day of dosing to confirm study eligibility.

Each subject participated in 3 treatment periods, each of 28 days duration: OC alone, OC 1 hour before exenatide, and OC approximately 30 minutes after exenatide. Exenatide was self-administered 15 minutes prior to the morning and evening meals at 5 μg BID on Days 1 through 4 and increased to 10 μg BID on Days 5 through 22. Subjects received a single dose of OC on Day 8 of each treatment period; dosing was omitted on Day 9 to allow for single-dose PK sampling. Subsequently, once-daily doses of OC were resumed on Days 10 through 28. Given that exenatide was administered 15 minutes before meals, OC was administered either 75 minutes before the meal (ie, 1 hour before exenatide) or 15 minutes after the meal (ie, 30 minutes after exenatide), depending on the treatment period. In the OC alone arm, all multiple OC doses and the majority of the single OC doses were given approximately 75 minutes before the meal.

During each treatment period after the first dose of the OC (Day 8), venous blood samples (4 mL each) were taken pre-dose, and at 0.5, 1, 1.5, 2, 2.5, 3, 3.5, 4, 4.5, 5, 6, 8, 10, 12, 16, 24 and 48 hours post-dose. Blood samples were also taken following multiple doses of the OC (Day 22) pre-dose, and at 0.5, 1, 1.5, 2, 2.5, 3, 3.5, 4, 4.5, 5, 6, 8, 10, 12, 16 and 24 hours post-dose. At a minimum, subjects were admitted to the Clinical Research Unit (CRU) on Day 7, resident on Day 8, and discharged on Day 9, then admitted again on Day 21, resident on Day 22, and discharged on Day 23. Subjects were required to attend the CRU on Days 10 and 28 as outpatients. At the investigator's discretion, subjects could be resident in the CRU or attend as outpatients after the first exenatide dose (Day 1) and upon dose increases to 10 μg (Day 5).

### Bioanalytical methods

Human plasma PK samples obtained during this study were analysed at PPD, Richmond, VA, USA. The samples were analysed for EE and LV using validated liquid chromatography with tandem mass spectrometric methods [15]. The lower limit of quantification was 2.00 pg/mL for EE and 50.0 pg/mL for LV; the upper limit of quantification was 200 pg/mL for EE and 12500 pg/mL for LV and. The intra-assay accuracy (% relative error) during partial validation ranged from -4.24% to 0.992% for LV and from 0.233% to 2.96% for EE. The intra-assay precision (% relative standard deviation) during partial validation ranged from 6.30% to 8.67% for LV and from 4.63% to 12.1% for EE.

### Pharmacokinetic assessments

Plasma EE and LV pharmacokinetics were characterised by noncompartmental methods of analysis using WinNonLin Professional Version 5.0.1 (Pharsight, Cary, NC). Plasma concentrations for each OC component were plotted semi-logarithmically against time following single (Day 8) or multiple doses (Day 22). The maximum concentration after single or multiple doses (C_max _or C_max, ss_) and the corresponding time of maximum concentration (T_max _or T_max, ss_) were identified from the observed data. After a single dose, the area under the concentration-time curve up to the last sampling time point (AUC_0-t, last_) was calculated and extrapolated to infinity (AUC_0-∞_) using the log-linear trapezoidal rule. Following multiple-dose administration of the OC, the area under the curve over the 24-hour post-dose interval (AUC_0-τ, ss_) was calculated on Day 22. Additionally, concentrations were tabulated at the 24-hour post-dose scheduled time points following single and multiple doses. These 24-hour post-dose concentrations are referred to as daily trough concentrations in the remainder of the document.

### Statistical methods

The statistical analysis included all data from subjects who received at least 1 dose of drug, and who had evaluable PK data. The primary PK parameters analysed statistically for EE and LV were AUC_0-∞ _and C_max _following single-dose administration (Day 8) and AUC_0-τ, ss _and C_max, ss _following multiple-dose administration (Day 22). In addition, OC trough concentrations on Day 8 and Day 22 were analysed. PK parameters were log-transformed (base e) prior to analysis. Single- (Day 8) and multiple-dose (Day 22) PK profiles of EE and LV were assessed separately. A linear mixed-effects model was applied that included subject as a random effect, and treatment, period, and sequence as fixed effects. The differences between treatments and the control (OC alone) were back-transformed to yield the ratio of the LS geometric mean for each PK parameter relative to the control treatment, and the corresponding 90% CI. An interaction was concluded when the 90% CI for the ratio of the LS geometric mean was not contained within the pre-specified interval (0.80, 1.25). Inter-and intra-subject variability estimates were derived from the mixed-effects model. T_max _was analysed separately for Day 8 and Day 22 using the nonparametric Wilcoxon rank sum test.

### Safety assessments

Safety was assessed by recording spontaneously reported adverse events and was evaluated at scheduled intervals by physical examination, vital sign measurement (including sitting blood pressure and heart rate), body weight assessments, clinical laboratory tests (including serum biochemistry, hematology, and urinalysis), and 12-lead electrocardiogram recordings.

## Results

### Subjects

A total of 38 healthy female subjects entered the study, 32 of these subjects were randomly assigned to 1 of the 3 sequences. Of the 38 subjects who entered the study, 20 completed the 3 treatment periods, and 18 subjects were withdrawn from the study. The mean (SD) age, weight and body mass index (BMI) for the 32 subjects assigned to treatment were 28 (6.8) years, 69.0 (9.3) kg and 25.1 (3.2) kg/m^2^, respectively. The majority (n = 31) of subjects were Caucasian. Twelve subjects were smokers.

Six subjects were withdrawn during the lead-in period, prior to being randomly assigned to a sequence. Four of these 6 subjects withdrew their consent during the run-in period because they were unable to participate on the required study dates. One subject was withdrawn due to appendicitis. One subject was withdrawn due to protocol non-compliance. The other 12 subjects were withdrawn after being assigned to treatment: 10 subjects due to adverse events and 2 subjects due to withdrawal of consent. Two of the 12 subjects were withdrawn when OC was given alone, 5 when OC was given 1 hour before exenatide, and 5 when OC was given 30 minutes after exenatide.

### Safety and tolerability

No break-through bleeding was reported during the study. The incidence of adverse events considered to be related to OC was generally similar across all treatments. An increase in the incidence of adverse events overall was observed with concomitant administration of exenatide and OC compared to administration of OC alone. The majority of subjects experienced adverse events considered to be related to exenatide. Overall, nausea and vomiting were reported by 91% and 81% of subjects, respectively. All cases of nausea were mild or moderate in severity. One case of vomiting was considered to be severe. Twenty subjects received concomitant medication for the treatment and/or prophylaxis of nausea and vomiting, and 6 subjects were withdrawn from the study due to mild or moderate nausea or vomiting. The incidence of vomiting was higher when the OC was administered 30 minutes after exenatide (74%), compared to 58% when administered 1 hour before exenatide; however, these incidences were not statistically different.

Seventeen subjects (53%) in the study reported skin-related adverse events, including injection-site rash (11 subjects) and skin rash (8 subjects). The skin-related adverse events were considered by the investigator to be related to exenatide in all but one of the cases. Two subjects were withdrawn from the study due to rash.

There were no clinically important trends in the serum biochemistry, hematology, urinalysis, blood pressure, heart rate, or 12-lead electrocardiogram data from baseline in each treatment period to follow-up.

### Pharmacokinetics

For subjects who discontinued the study prior to completion of all 3 treatment periods, pharmacokinetics (PK) data generated in other completed periods were included in the PK assessments. Although some subjects experienced vomiting on PK-assessment days, none of these data were excluded from analyses as their concentrations at a scheduled time point were within the pre-specified outlier threshold of 3 standard deviations from the mean for the remainder of concentrations at that time point.

### Single-dose pharmacokinetics

**Figure 1:**
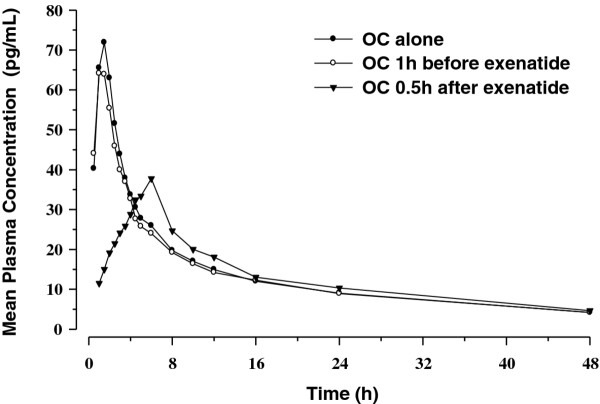
**Mean single-dose plasma concentration-time profiles for ethinyl estradiol**.

Mean plasma concentration time profiles following single-dose administration EE and LV are shown in Figures 1 (EE) and 2 (LV). Mean plasma concentration time profiles associated with OC given 1 hour before exenatide were similar to those observed with OC alone. Mean plasma EE and plasma LV concentration time profiles following OC administration 30 minutes after exenatide were characterised by a reduced C_max _and delayed T_max_.

**Figure 2:**
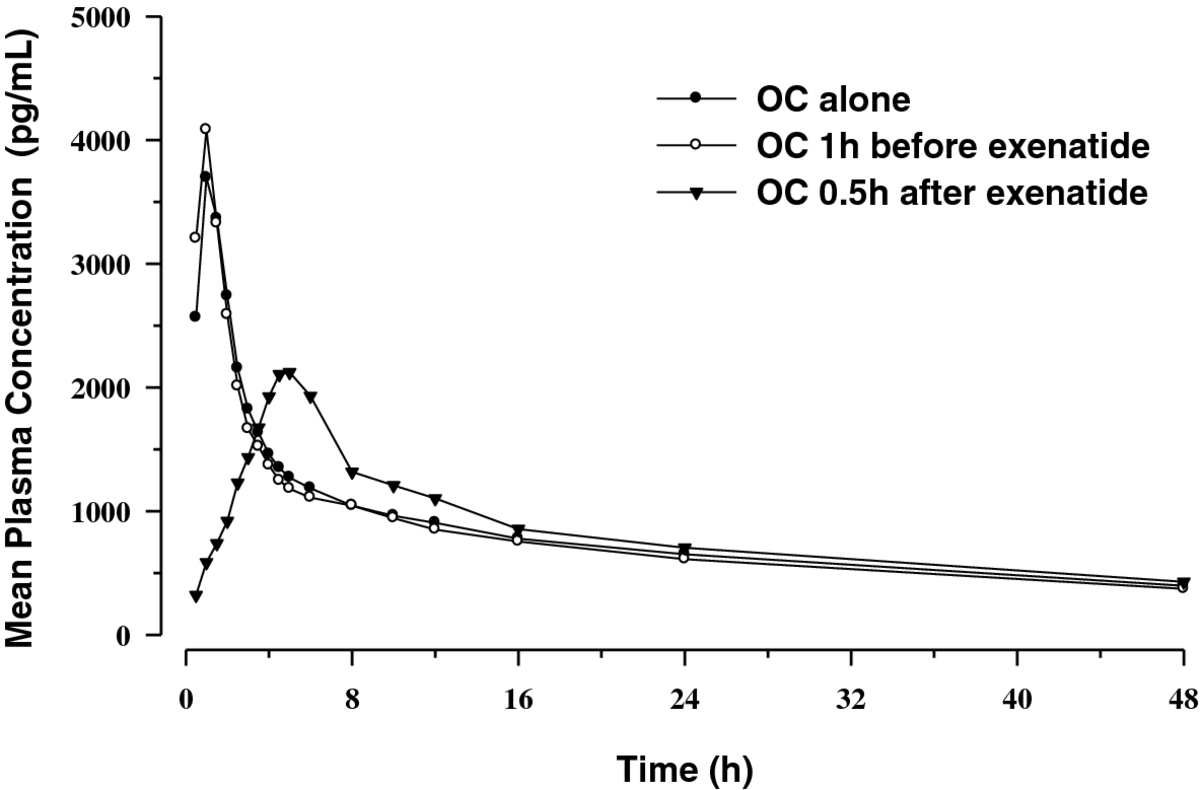
**Mean single-dose plasma concentration-time profiles for levonorgestrel**.

Statistical comparisons for single-dose PK parameters are shown in Table 1 (EE) and Table 2 (LV). Consistent with the graphical evaluations, concomitant exenatide administration did not alter the bioavailability (as measured by AUC_0-∞_) of EE or LV, as the 90% confidence intervals (CI) for the ratios of the least-squares (LS) geometric means were contained within the pre-specified "no effect" range (0.8 to 1.25). Administration of OC 1 hour before exenatide did not result in a change in C_max _for either EE or LV, but administration of OC 30 minutes after exenatide resulted in reductions in C_max _of 46% and 41% for EE and LV, respectively, compared to OC given alone. These reductions in C_max _were accompanied by median T_max _increases of 4.05 and 3.75 hours for EE and LV, respectively. No differences in trough concentrations of either EE or LV were observed when OC was administered 1 hour before exenatide. Increases in trough concentrations for both EE (24%) and LV (15%) were observed when OC was administered 30 minutes after exenatide.

**Table 1:** Ethinyl Estradiol Single- and Multiple-dose Pharmacokinetics: Comparison of Mean Pharmacokinetic Parameters Across Treatments

		Single Dose		Multiple Dose	
		**LS Geometric Mean**	**Comparison to OC alone: Ratio (90% CI)**	**LS Geometric Mean**	**Comparison to OC alone: Ratio (90% CI)**

**AUC (pg·h/mL)**	OC alone	718.89	-	761.06	-

	1 h before exenatide	691.69	0.96 (0.91, 1.02)	716.70	0.94 (0.88, 1.01)

	0.5 h after exenatide	692.56	0.96 (0.91, 1.02)	734.01	0.96 (0.90, 1.04)

**C_max _(pg/mL)**	OC alone	72.18	-	102.15	-

	1 h before exenatide	65.49	0.91 (0.83, 0.99)	87.09	0.85 (0.78, 0.93)

	0.5 h after exenatide	38.64	0.54 (0.49, 0.58)	56.32	0.55 (0.50, 0.60)

**24-h concentration (pg/mL)**	OC alone	8.27	-	14.64	-

	1 h before exenatide	8.13	0.98 (0.92, 1.05)	15.03	1.03 (0.95, 1.11)

	0.5 h after exenatide	10.25	1.24 (1.16, 1.33)	17.52	1.20 (1.10, 1.30)

**T_max _median (range) (h)**	OC alone	-	1.50 (0.50-2.50)	-	-

	1 h before exenatide	-	1.50 (0.50-2.00)	-	-

	0.5 h after exenatide	-	6.00 (2.00-6.13)	-	-

**t_1/2 _geometric mean (range) (h)**	OC alone	-	19.5 (13.8-32.1)	-	-

	1 h before exenatide	-	18.9 (14.0-30.1)	-	-

	0.5 h after exenatide	-	17.4 (8.78-31.9)	-	-

**Table 2:** Levonorgestrel Single- and Multiple-dose Pharmacokinetics: Comparison of Mean Pharmacokinetic Parameters Across Treatments

		Single Dose		Multiple Dose	
		**LS Geometric Mean**	**Comparison to OC alone: Ratio (90% CI)**	**LS Geometric Mean**	**Comparison to OC alone: Ratio (90% CI)**

**AUC (pg·h/mL)**	OC alone	55698.79	-	72974.62	-

	1 h before exenatide	53530.64	0.96 (0.90, 1.03)	72952.67	1.00 (0.92, 1.09)

	0.5 h after exenatide	60591.89	1.09 (1.01, 1.17)	76344.29	1.05 (0.96, 1.14)

**C_max _(pg/mL)**	OC alone	3882.56	-	6598.95	-

	1 h before exenatide	4061.86	1.05 (0.94, 1.16)	6657.22	1.01 (0.92, 1.10)

	0.5 h after exenatide	2284.25	0.59 (0.53, 0.65)	4800.68	0.73 (0.67, 0.79)

**24-h concentration (pg/mL)**	OC alone	600.88	-	2136.51	-

	1 h before exenatide	571.47	0.95 (0.89, 1.02)	2173.05	1.02 (0.93, 1.11)

	0.5 h after exenatide	691.10	1.15 (1.07, 1.23)	2367.68	1.11 (1.01, 1.21)

**T_max _median (range) (h)**	OC alone	-	1.00 (0.50-2.07)	-	-

	1 h before exenatide	-	0.92 (0.50-1.50)	-	-

	0.5 h after exenatide	-	4.75 (3.00-6.02)	-	-

**t_1/2 _geometric mean (range) (h)**	OC alone	-	33.6 (20.0-78.5)	-	-

	1 h before exenatide	-	32.1 (19.8-55.7)	-	-

	0.5 h after exenatide	-	32.6 (17.9-72.4)	-	-

### Multiple-dose pharmacokinetics

**Figure 3:**
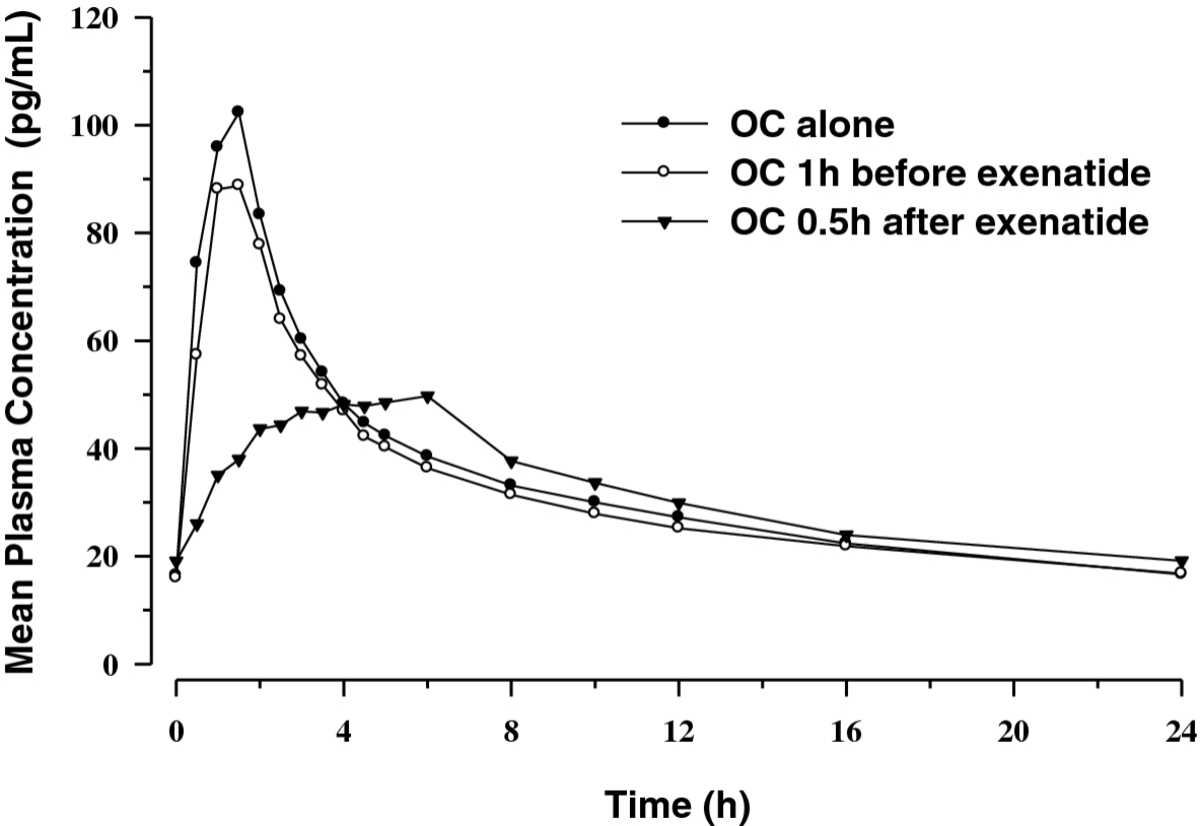
**Mean steady-state plasma concentration-time profiles for ethinyl estradiol**.

Mean plasma concentration time profiles following repeated daily dosing (Day 22) are presented in Figures 3 (EE) and 4 (LV). Similar to the single-dose PK evaluations, results from the multiple-dose PK assessments demonstrated that administration of OC 30 minutes after exenatide reduced C_max _and delayed T_max _for both EE and LV compared with OC given alone. As with the single-dose period, PK differences following repeated daily dosing were mainly evident in the absorptive phase. When OC was given 1 hour before exenatide, no changes in the OC PK profiles were observed.

**Figure 4:**
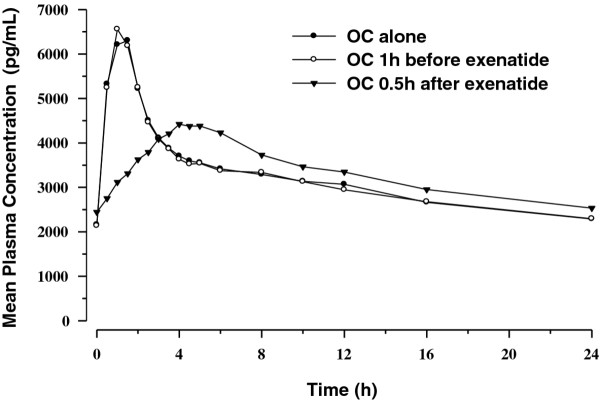
**Mean steady-state plasma concentration-time profiles for levonorgestrel**.

Between-treatment statistical comparisons for multiple-dose PK parameters are shown in Table 1 (EE) and Table 2 (LV). As with the single-dose data, regardless of timing of exenatide administration relative to OC dosing, no changes in bioavailability (AUC_0-τ, ss_) of either EE or LV were observed after concomitant repeated daily administration of OC and exenatide. Compared to OC given alone, repeated daily administration of OC 1 hour before exenatide resulted in a small reduction in EE C_max _of approximately 15% but did not alter LV C_max_. Reductions in C_max _were more notable when the OC was administered 30 minutes after exenatide. Repeated daily doses of OC given 30 minutes after exenatide reduced EE and LV C_max _by 45% and 27%, respectively, compared with OC given alone. Median T_max _was also delayed by 3 hours and 3.5 hours for EE and LV, respectively, compared with OC alone. As observed in the single-dose results, increases in trough concentrations for both EE (20%) and LV (11%) were observed when OC was administered 30 minutes after exenatide. No differences in trough concentrations of EE or LV were observed when OC was administered 1 hour before exenatide.

## Discussion

In this pharmacokinetic drug-interaction study, co-administration with exenatide did not affect mean plasma AUC of EE and LV under single- or multiple-dose conditions. Furthermore, no decreases in trough concentrations were observed. Additionally, no substantive changes in PK profiles were observed when the OC was administered 1 hour before exenatide. A small effect was observed (90% CI; 0.78, 0.93) in C_max _when OC was administered alone, however, as the lower confidence bound is close to 0.8, this shift is not likely to be of clinical relevance. Reductions in peak OC concentrations, accompanied by delayed T_max_, were observed with OC administered 30 minutes after exenatide. This effect of exenatide on OC absorption would be expected, due to its action to slow gastric emptying, and is consistent with prior drug-drug interaction studies of exenatide with other orally administered drugs [8-12].

Drug-drug interaction studies with oral contraceptives are generally conducted to understand the potential for concomitantly administered drugs to induce or inhibit cytochrome P450 isoenzyme (CYP) mediated oxidative metabolism of EE [16]. While EE is metabolised by both CYP3A-mediated oxidative metabolism and Phase II metabolism, including glucuronidation and sulfation, the most clinically relevant metabolic pathway is induction or inhibition of CYP3A. Drugs that decrease EE bioavailability via CYP3A induction may potentially result in reduced OC efficacy. The observation that OC AUC concentrations were unaltered in this study confirmed that exenatide does not induce CYP3A. However, the clinical relevance of C_max _reductions seen in the study (up to 46% for EE and 41% for LV) requires additional consideration.

Reports of large inter-subject variability in concentrations of OCs, with several-fold differences in serum concentrations likely due to inter-individual differences in first-pass metabolism, have been described in the literature. Goldzieher et al. [17] have reported that differences in EE concentrations have been shown to vary between ethnic groups, as well as across study sites and, even for a given individual, EE AUCs can vary by almost a factor of 4. This same study group has also reported the existence of high intra- and inter-subject variability in the pharmacokinetics of progestins such as LV [17]. Thus, the magnitude of C_max _reduction observed in the present study was likely within the inherent PK variability of the OC components.

The current study did not measure OC pharmacodynamics (eg, follicle-stimulating hormone or luteinizing hormone concentrations); therefore, a direct within-study clinical relationship with the observed C_max _reduction cannot be derived. Importantly, no break-through bleeding was reported. Break-through bleeding may be associated with low concentrations of estrogen-progestin [16,18].

A review of prescription labeling indicates that drug interactions with OCs are deemed to be clinically important, and dosage adjustments are thereby recommended, only when associated with a significant reduction in OC AUC. However, there does not appear to be a well-accepted minimum threshold concentration for pharmacological activity. Importantly, in this study OC AUC was unchanged. Furthermore, trough concentrations did not decrease in the presence of exenatide suggesting that sub-therapeutic concentrations were unlikely.

In the absence of conclusive literature on the exposure-efficacy relationships of OCs, other aspects of OC PK/PD were considered to help understand the possible clinical relevance of the C_max _decrease observed in the present study. In food-effect studies, OC C_max _reductions of up to 40% are commonly observed without changes in AUC. Despite this potential effect of food on C_max_, OCs are generally recommended to be taken without regard to food [19], suggesting that PK changes observed in this study are not likely to be clinically relevant. In the current study, the effect of food consumption on the OC PK cannot be clearly differentiated from the effects of exenatide; however, these data reflect conditions under which the 2 drugs are likely to be co-administered, given that exenatide is to be administered within an hour of meals. Thus, in consideration of indirect evidence from food-effect studies, the large inherent variability in OC concentrations, and the fact that product labeling suggests changes in OC dosage only in the presence of large changes in OC AUC alone, we conclude that the PK changes observed in the present study are not likely to have clinical implications.

Concomitant administration of exenatide and a combination OC to healthy female subjects resulted in a high incidence of gastrointestinal adverse events. This study used 4-day dose initiation at 5 μg BID, rather than 4 weeks as recommended for exenatide dosing [20], and this may have contributed to the poor tolerability. Furthermore, cross-study comparisons have suggested that exenatide administration may result in a higher incidence of gastrointestinal adverse events in healthy subjects compared with patients with type 2 diabetes [8]. More specifically, the high incidence of nausea and vomiting observed in the present drug-drug interaction study has not been observed in large clinical trials of exenatide-treated patients with type 2 diabetes [21-24]. Additionally, although there were no unexpected adverse events observed in this study, the incidence of skin-related adverse events among exenatide-treated subjects (53%) was higher than the incidence observed in previous clinical studies [21-24].

## Conclusions

This study evaluated the potential effect of subcutaneously administered exenatide (10 μg BID) on the single and multiple doses pharmacokinetics of a combination OC (EE/LV). No pharmacokinetic interaction was observed when the OC was administered an hour prior to exenatide treatment. However, administration of the OC 30 minutes after exenatide therapy was associated with a reduced C_max _with a delayed T_max _of EE and LV. The observed reduction in C_max _is likely of limited importance as co-administration of exenatide did not cause significant changes in the overall bioavailability of EE or LV after single or multiple doses. For oral medications that are dependent on threshold concentrations for efficacy, such as contraceptives and antibiotics, patients should be advised to take those drugs at least 1 hour before exenatide injection.

## Competing interests

This study was sponsored by Amylin Pharmaceuticals, Inc. and Eli Lilly and Company. Sponsors were involved in the study design, protocol development, and the collection, review, and analysis of the data. Authors are employees of Eli Lilly and Company.

## Authors' contributions

PAK, MES, HL, MM, and KM participated in the design of the study. PAK, HL, MES, and JN have been involved in drafting the manuscript. All authors have participated in the analysis and interpretation of data, and the critical revision for important intellectual content. All authors approved the version to be published.

## Pre-publication history

The pre-publication history for this paper can be accessed here:

http://www.biomedcentral.com/1472-6904/12/8/prepub
